# Supplementary material for: A structural basis for the diverse linkage specificities within the ZUFSP deubiquitinase family
Source: Nat Commun. 2022 Jan 20;13:401. doi: 10.1038/s41467-022-28049-6 (PMC8776766; doi:10.1038/s41467-022-28049-6)
Supplement: Supplementary file 1 — Supplementary information [file 41467_2022_28049_MOESM1_ESM.pdf]

Supplementary Information

A structural basis for the diverse linkage specificities within the ZUFSP deubiquitinase family

Hermanns et al.

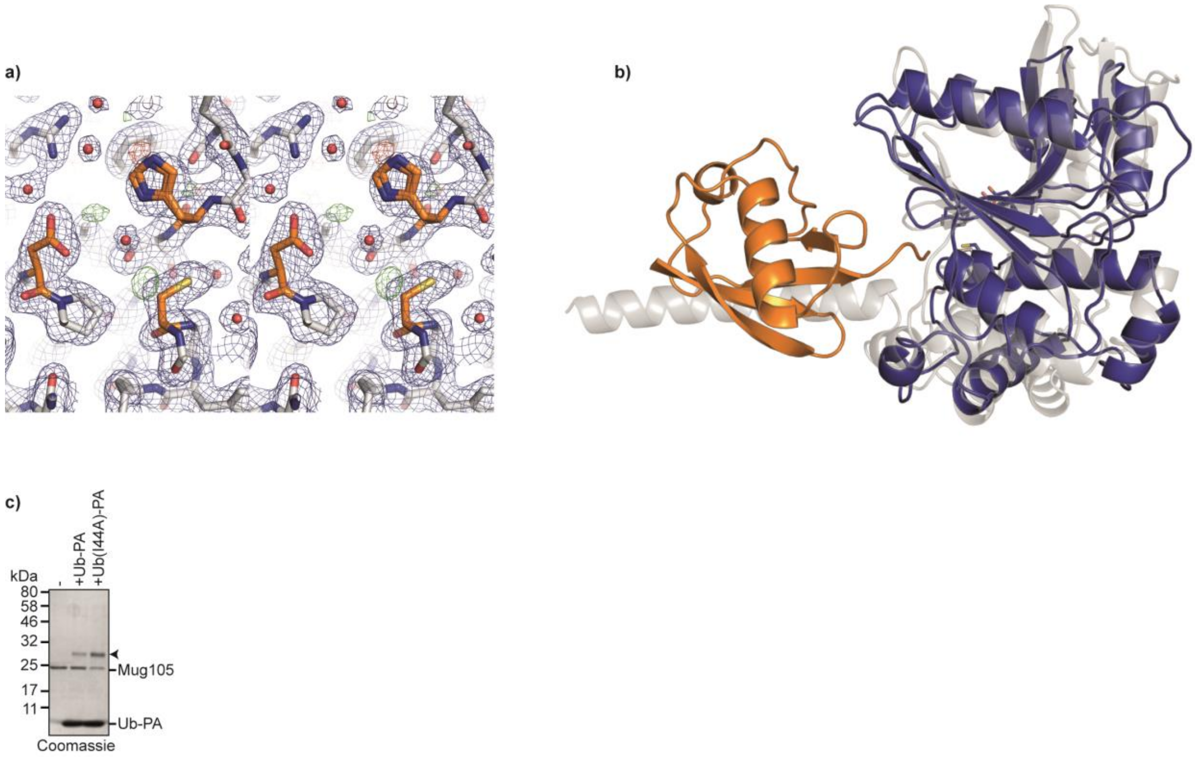

Supplementary Figure 1: Ubiquitin binding interface of Mug105 a) Stereo view on the active site of Mug105. The catalytic residues are highlighted in orange and the remaining residues are colored in light grey. The experimental electron density map (2mFo-DFc) is shown as blue mesh at a contour level of 1 σ and the difference density map (mFo-DFc) is shown in green and red at a contour level of 3 σ and - 3 σ respectively. **b)** Ubiquitin (orange) derived from the ZUP1 structure (6EI1, transparent, light grey)is modelled into the Mug105 structure (blue) by structural superposition of Mug105 and ZUP1. **c)** Activity based probe reaction of Mug105 with Ub-PA or Ub^{I44A}-PA. Black arrowhead mark the shifted band after reaction. Source data are provided as a Source Data file.

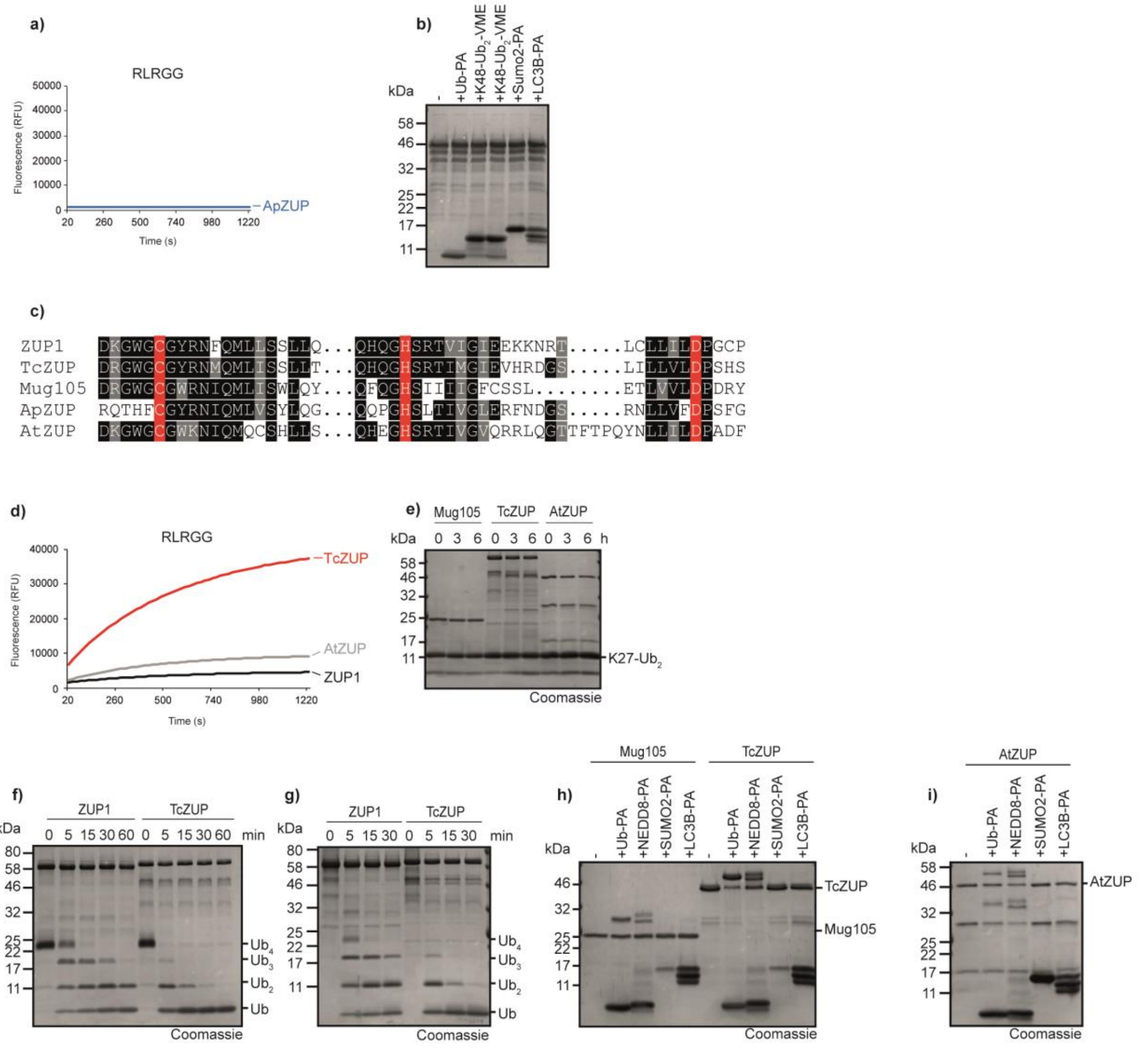

Supplementary Figure 2: Activities in the ZUFSP family. a) Activity of the ZUP homolog from *Aureobasidium pullulans* (ApZUP) against RLRGG-AMC. The RFU values shown are the means of triplicates. **b)** Lack of reactivity of ApZUP with various Ub/UbL activity-based probes. **c)** Alignment of sequence blocks surrounding the active site residues of ZUFSP family members used in this study. Invariant and conservatively replaced residues are shown on black or grey background, respectively. Active site residues are shown on red background. **d)** Activity of ZUP homologues from *A. thaliana* (AtZUP) and *T. castaneum* (TcZUP) against RLRGG-AMC. A lower DUB concentration (0.1 µM) was used for all DUBs in order to highlight the high activity of TcZUP. The RFU values shown are the means of triplicates. **e)** Activity of ZUP homologues Mug105, TcZUP or AtZUP against K27-linked di-ubiquitin. **f,g)** Activity of ZUP1 and TcZUP against K63-linked Ub_4 (f) or Ub_{6+} (g) chains. **h,i)** Activity based probe reaction of ZUP1 homologues Mug105, TcZUP (h) or AtZUP (i) with a set of UbL-PAs. Source data are provided as a Source Data file.

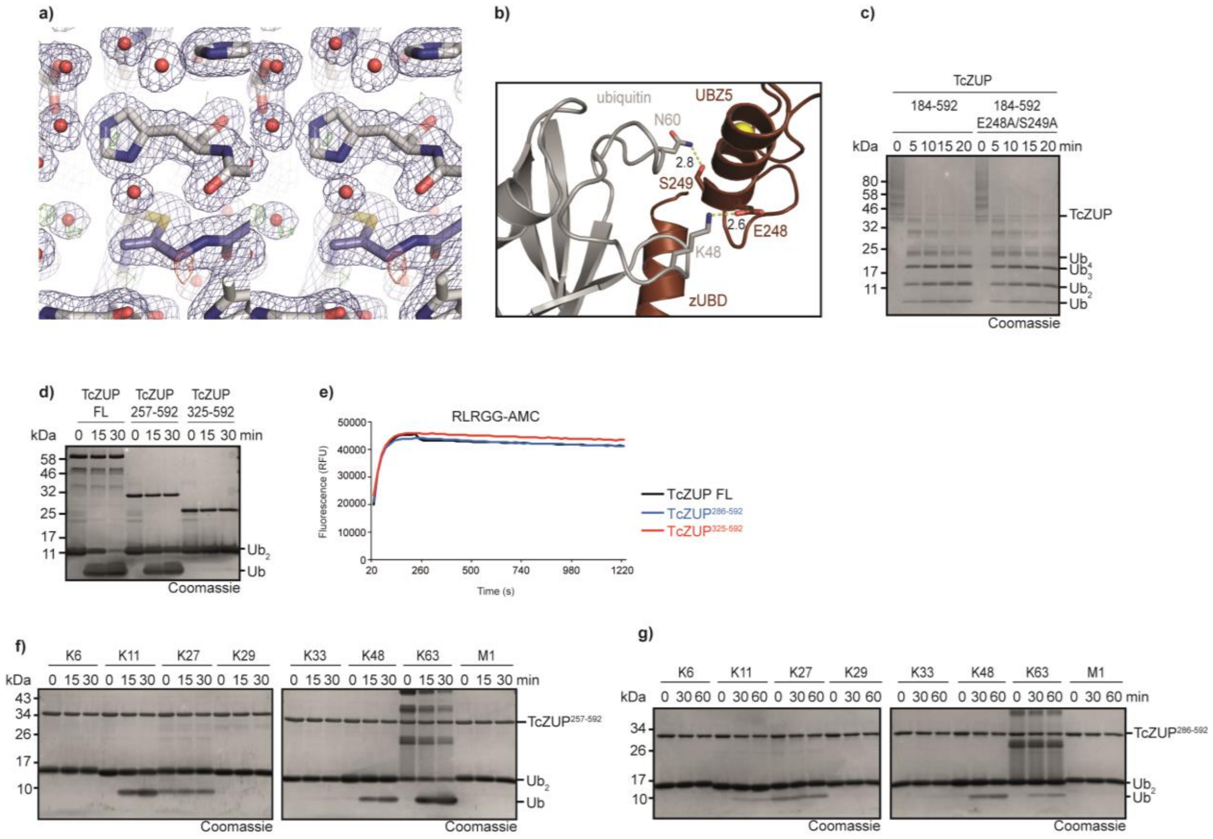

Supplementary Figure 3: TcZUP Glu-248/Ser-249 are not involved in ubiquitin binding. **a)** Stereo view on the active site of TcZUP. TcZUP residues are colored in light grey and the ubiquitin c-terminus is colored in blue. The experimental electron density map (2mFo-DFc) is shown as blue mesh at a contour level of 1 σ and the difference density map (mFo-DFc) is shown in green and red at a contour level of 3 σ and - 3 σ respectively. **b)** TcZUP (brown) and ubiquitin (light grey) are shown in cartoon representation. Interactions between the UBZ5 of TcZUP and ubiquitin are indicated by yellow dotted lines and the involved residues are highlighted as sticks. **c)** Activity of the $TcZUP^{184-592}$ UBZ5 mutant E248A/S249A against K63-linked Ub_{6+} chains. **d)** Activity of the TcZUP truncations $TcZUP^{257-592}$ and $TcZUP^{325-592}$ against K63-linked di-ubiquitin. **e)** Activity of the TcZUP truncations $TcZUP^{286-592}$ (α2/3 + cat. domain) or $TcZUP^{325-592}$ (cat. domain only) against RLRGG-AMC. The RFU values shown are the means of triplicates. **f,g)** Linkage specificity analysis of TcZUP truncations containing or lacking the zUBD region. A panel of di-ubiquitin was treated with $TcZUP^{257-592}$ (f) or $TcZUP^{286-592}$ (g) for the indicated time points. Source data are provided as a Source Data file.

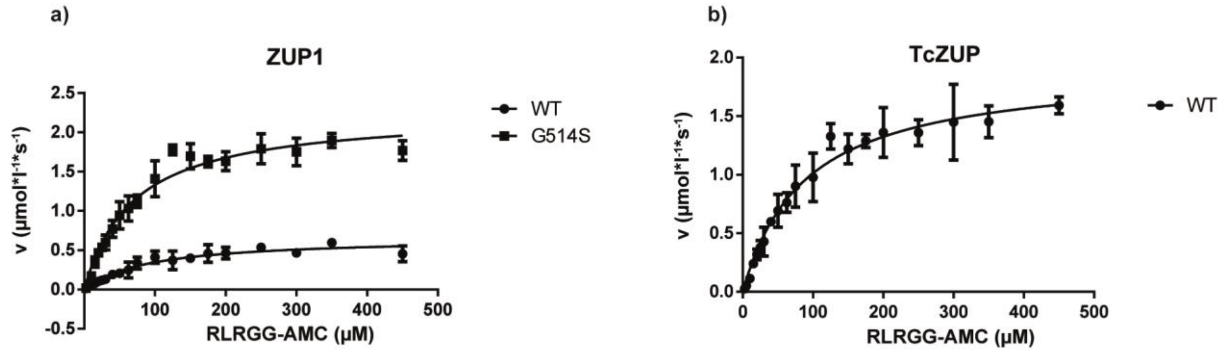

c)

	$k_{cat}(s^{-1})$	K_m(µM)
ZUP1	6.6 ± 0.4	91.4 ± 14.3
ZUP1 G514S	22.6 ± 0.7	69.9 ± 6.6
TcZUP	95.6 ± 3.7	89 ± 9.3

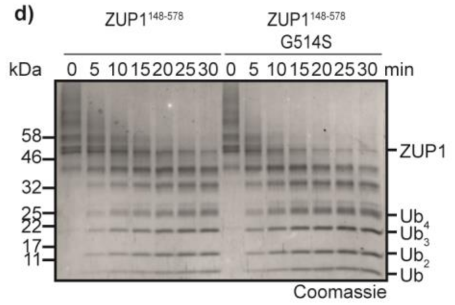

Supplementary Figure 4: ZUP1^{G514S} mutant increases activity by a k_{cat} effect. a,b) Steady state kinetics using RLRGG-AMC as substrate at varying concentrations. The mean initial rates of three independent experiments were plotted against the substrate concentration and a Michaels-Menten curve was fitted to the experimental data. Error bars represent the standard deviation of three independent measurements, each of them using either 100 nM ZUP1 (a) or 20 nM TcZUP (b). **c)** Kinetic parameters derived from Michaels-Menten curves (a,b) for ZUP1, ZUP1^{G514S} and TcZUP. The parameters are presented ± the standard error. **d)** Activity of the ZUP1$^{148\text{-}578}$ oxyanion hole mutant G514S against K63-linked Ub_{6+} chains. Source data are provided as a Source Data file.

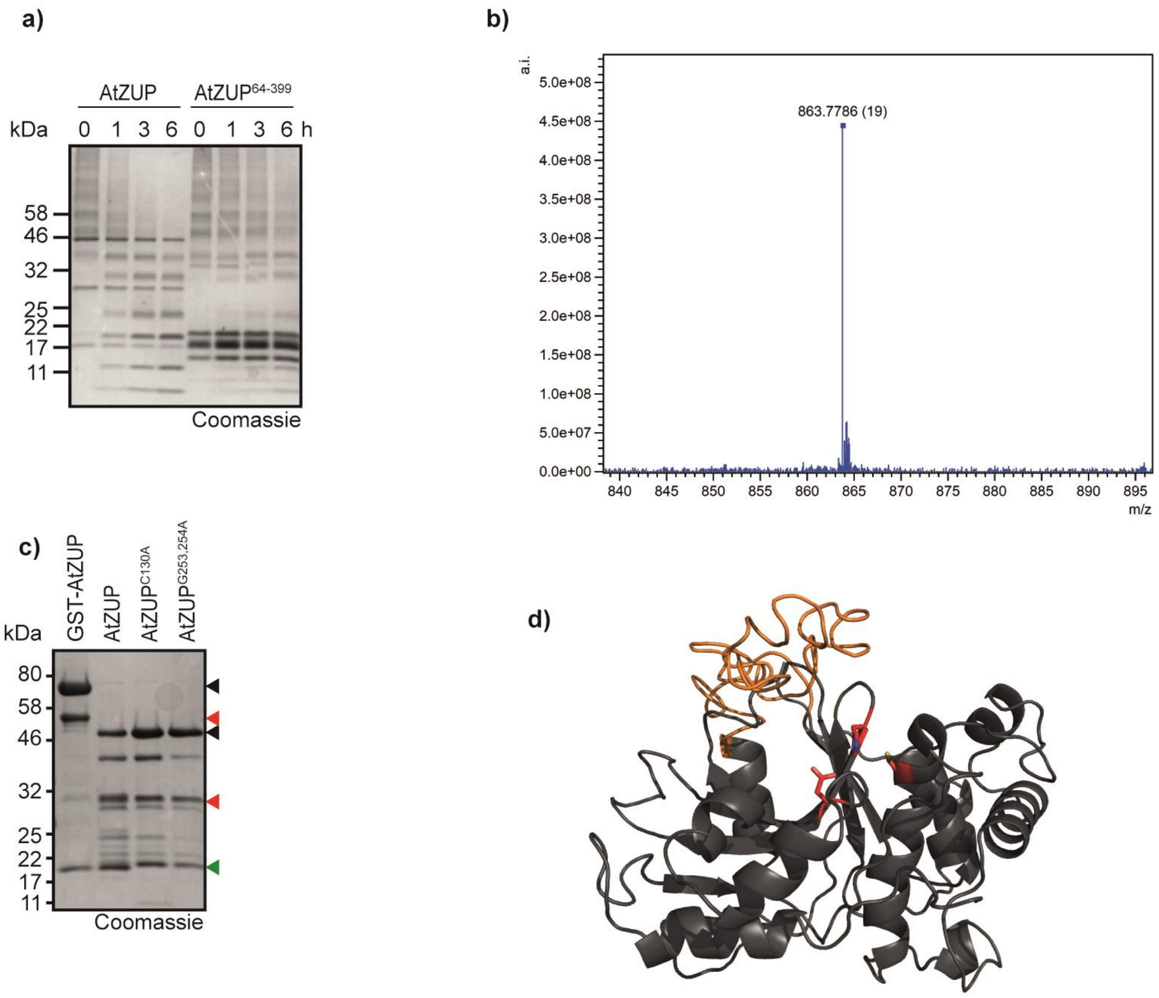

Supplementary Figure 5: Activity of AtZUP truncations and AtZUP intra-loop cleavage. **a)** Activity of the UBZ-lacking truncation AtZUP$^{64\text{-}399}$ against K63-linked Ub$_{6+}$ chains. **b)** Intact mass analysis of FL AtZUP, showing the fragmentation. Only one of the resulting fragments could be measured and is shown as intensity (a.i.) over mass (m/z). The determined mass of 863.78 corresponds to 19 charges and can be deconvoluted to a mass of 16392.66 Da. This mass fits to the C-terminal fragment of AtZUP1 starting at Ser-255 with an expected monoisotopic mass of 16392.32 Da. **c)** Fragmentation is observed in bacterially expressed GST-AtZUP fusion protein, AtZUP after GST cleavage, and the AtZUP mutants C130A and G253A/G254A. Black arrowheads indicate full-length proteins, red and green arrowheads indicate the larger N-terminal and the smaller C-terminal fragment, respectively. **d)** A model of AtZUP was generated by I-TASSER using ZUP1 and Mug105 as a model [35]. The catalytic core of AtZUP is shown in cartoon representation and colored dark grey. The active site residues are shown as sticks and colored red. The long flexible loop (residues 219 to 275) is colored orange. Source data are provided as a Source Data file.

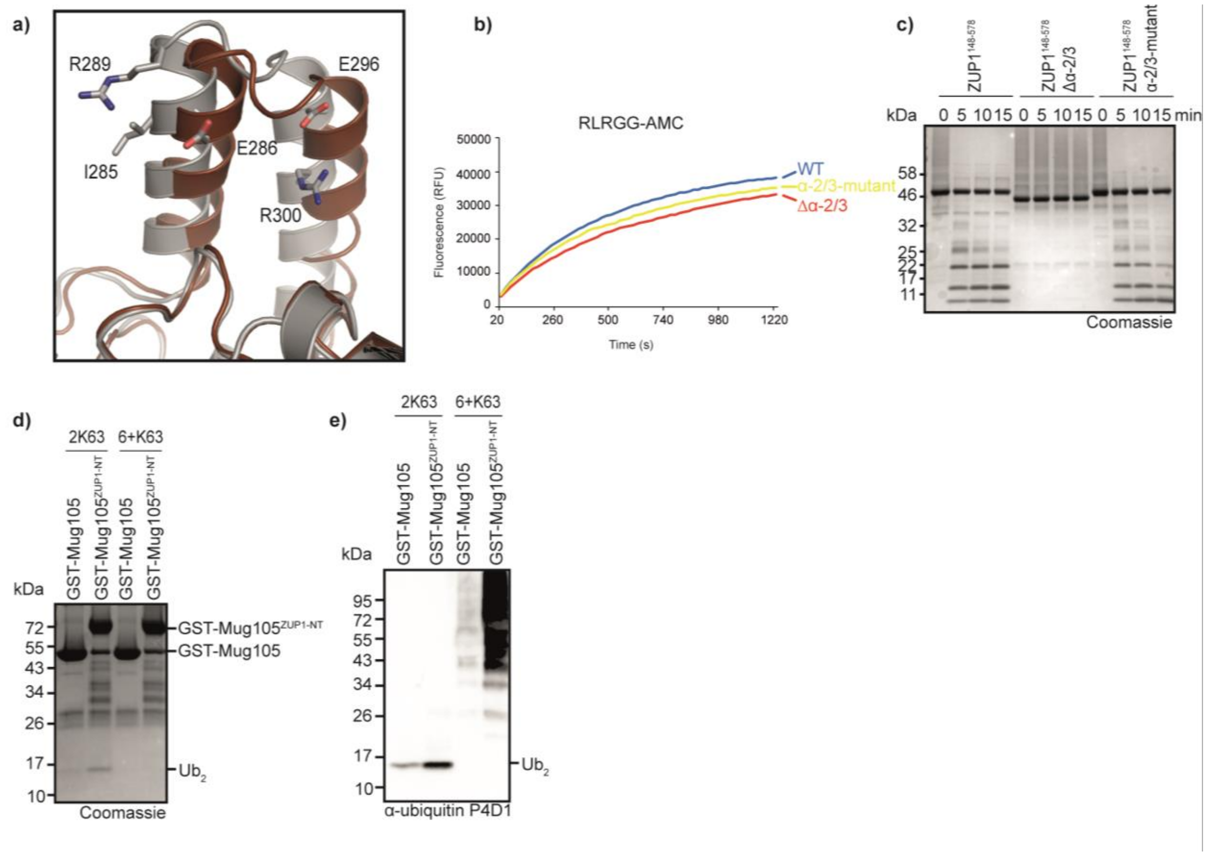

Supplementary Figure 6: Mutations of the α-2/3 helices do not impair ZUP1 activity. **a)** Superposition of the α-2/3 helices from ZUP1 (light grey) and TcZUP (brown). Five surface-exposed residues facing towards the active site are highlighted as sticks and mutated to alanine in the following experiments. **b,c)** Activity assays of the quintuple $ZUP1^{148\text{-}578}$ α-2/3 mutant (I285A, E286A, R289A, E296A, R300A) against RLRGG-AMC (b) or K63-linked Ub_{6+} chains (c). **d,e)** Pull-down analysis of Mug105 and the chimeric $Mug105^{ZUP1\text{-}NT}$ against K63-linked di-ubiquitin (Ub_2) or longer chains (Ub_{6+}). Eluted proteins were separated by SDS-PAGE and stained by Coomassie staining (d) or immuno-stained with α-ubiquitin P4D1 antibody (e). Source data are provided as a Source Data file.

Supplementary Table1: Primers used in this study

primer name	sequence in 5'- 3'
pOPINS AtZUP 1 fwd	GCGAACAGATCGGTGGTATGTCAGCTTTGTGTCCTGTCTGCAATCTCACGC
pOPINK AtZUP 1 fwd	AAGTTCTGTTTCAGGGCCCGATGTCAGCTTTGTGTCCTGTCTGCAATCTCACGC
pOPINS AtZUP 64 fwd	GCGAACAGATCGGTGGTAACAACGTTGCCTCCTTGGTCCAGTTACAGACTAAG
pOPINS AtZUP 86 fwd	GCGAACAGATCGGTGGTTTGATTTGTTTGCTGAGGAACTGTCTGGAGTCAGAACTG
pOPINK AtZUP 394 rev	ATGGTCTAGAAAGCTTTAGAATTCAACAAAGTGACTATCAATGGTTTTGAGCTTCTCCAACTC
AtZUP C130A fwd	GAAGGAGGATAAAGGTTGGGGAGCTGGATGGAAGAACATTC
AtZUP C130A rev	GAATGTTCTTCCATCCAGCTCCCCAACCTTTATCCTCCTTC
AtZUP G253,254A fwd	GCTTTTCCCTTCCCACTTGCCGCCTTTTTAACCATATATC
AtZUP G253,254A rev	GATATATGGTTAAAAAGGCGGCAAGTGGGAAGGGAAAAGC
pOPINS ApZup 1 fwd	GCGAACAGATCGGTGGTATGGAGTGCCCTTTTCTTTGTGGTTTCCACACTCC
pOPINS ApZup1 449 rev	ATGGTCTAGAAAGCTTTACACAAGCACCTCGAACTCATCATACTTAGCTAATTGAGC
pOPINS TcZUP 1 fwd	GCGAACAGATCGGTGGTATGGCTAGCAACATCCCCGATTTGTTCTACTCCTG
pOPINS TcZUP 592 rev	ATGGTCTAGAAAGCTTTACCGGTCCTGCGGTATTCTCGTACCCC
pOPINS TcZUP36 fwd	GCGAACAGATCGGTGGTGGCAATGCCGTGTGCCCCTTTTGCGAG
pOPINS TcZUP133 fwd	GCGAACAGATCGGTGGTGGCTCCCCCTTGCGATCCAGTCTCG
pOPINS TcZUP184 fwd	GCGAACAGATCGGTGGTGGCACCACCAGCGAGATATACTCGTGTCC
pOPINS TcZUP218 fwd	GCGAACAGATCGGTGGTTCGCCTGCCAGTCCCCCAACACATTCG
pOPINS TcZUP 257 fwd	GCGAACAGATCGGTGGTCAGCCCGCCGACAGAGCAGCGTTG
pOPINS TcZUP 286 fwd	GCGAACAGATCGGTGGTAACTTCAGGGAACAGTCCGTTACGAACATGCAAAGAG
pOPINS TcZUP 325 fwd	GCGAACAGATCGGTGGTGACGATGGGAGCTCCATAACCAGGAGTATTGTG
Mug105 D89A fwd	CATAACCTTCGGCAGCTATACCTTTCATCCAAGCGCT
Mug105 D89A rev	AGCGCTTGGATGAAAGGTATAGCTGCCGAAGGTTATG

Mug105 E109A fwd	CGGTAAATAGGCTATAAACCGCAGTAGCACCAATCCATTTA
Mug105 E109A rev	TAAATGGATTGGTGCTACTGCGGTTTATAGCCTATTTACCG
Mug Q215A fwd	GTTTTGCTTAAAATGTACCAATTGAAATGCTGAGAATTTCAAAGATCGCTTTTTTCGC
Mug Q215A rev	GCGAAAAAAGCGATCTTTGAAATTCTCAGCATTTCAATTGGTACATTTTAAGCAAAAC
Mug105 D185S fwd	GAACACTCTGATAACGACTTGGATCAAGCACAACAAGAGTTTCTAAAGAA
Mug105 D185S rev	TTCTTTAGAAACTCTTGTTGTGCTTGATCCAAGTCGTTATCAGAGTGTTC
Mug105 D185G fwd	TTGAACACTCTGATAACGACCTGGATCAAGCACAACAAG
Mug105 D185G rev	CTTGTTGTGCTTGATCCAGGTCGTTATCAGAGTGTTCAA
Mug Q163A rev	GATAATGATAGAATGCCCTGCAAATTGTAAATAACATGGAGAAACATTGCTTGTATCA
Mug Q163A fwd	TGATACAAGCAATGTTTCTCCATGTTATTTACAATTTGCAGGGCATTCTATCATTATC
Mug W104A fwd	CTCAGTAGCACCAATCGCTTTACCATGCAAGTTGTCTCCTAA
Mug W104A rev	TTAGGAGACAACTTGCATGGTAAAGCGATTGGTGCTACTGAG
Mug1 ZUP1OV fwd	GGAAAAACAAAAACTATGTCAAAATGCTTGCAGCAGCTTAAACGACAACTTCAGC
ZUP1Q547A fwd	CCTCTACTGCCAATATCTGGTATGCCTTATGTTTTAAATTTCCCATAGATTTCC
ZUP1Q547A rev	GGAAATCTATGGGAAATTTAAAACATAAGGCATACCAGATATTGGCAGTAGAGG
ZUP1G514S fwd	CATTTCTCGAGAAGGACAGCTAGGATCAAGTATTAGTAAGCATAATGTTCGG
ZUP1G514S rev	CCGAACATTATGCTTACTAATACTTGATCCTAGCTGTCCTTCTCGAGAAATG
ZUP1Q489A fwd	TTCGACTGTGACCTGCATGCTGAAGATAGATAGGAGGTTTAGA
ZUP1Q489A rev	TCTAAACCTCCTATCTATCTTCAGCATGCAGGTCACAGTCGAA
ZUP1W423A fwd	TACTTCACATGCTCCAATCGCGGCCTTTGTTCCCTGTAAC
ZUP1W423A rev	GTTACAGGGAACAAAGGCCGCGATTGGAGCATGTGAAGTA
ZUP1G354P fwd	CCAACCTTTGTCGGGTAAAGATGAATGAAAGTGATCCACC
ZUP1G354P rev	GGTGGATCACTTTCATTCATCTTTACCCGACAAAGGTTGG
TcZUP S528G fwd	GTTGGGGGGAATGGCCCGGATCTAGCACTAG
TcZUP S528G rev	CTAGTGCTAGATCCGGGCCATTCCCCCCAAC
TcZUP Q503A fwd	TCCGGCTGTGACCTGCATGTTGTAAATACAGTGGTGGAACG
TcZUP Q503A rev	CGTTCCACCACTGTATTTACAACATGCAGGTCACAGCCGGA
TcZUP W443A fwd	GACTTCGGTGGCTCCAATCGCTTTGCGAGTATTAACAAGA
TcZUP W443A rev	TCTTGTTAATACTCGCAAAGCGATTGGAGCCACCGAAGTC
