# Supplementary material for: A structural basis for the diverse linkage specificities within the ZUFSP deubiquitinase family
Source: Nat Commun. 2022 Jan 20;13:401. doi: 10.1038/s41467-022-28049-6 (PMC8776766; doi:10.1038/s41467-022-28049-6)
Supplement: Supplementary file 4 — Source Data [file 41467_2022_28049_MOESM4_ESM.zip › Source Data.pdf]

Fig. 1F)

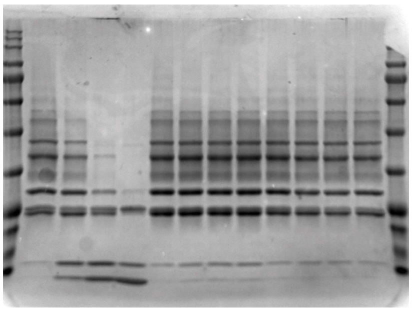

Fig. 1I)

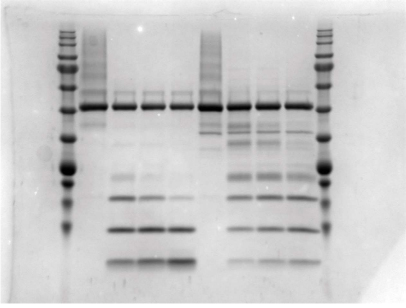

Fig. 1K)

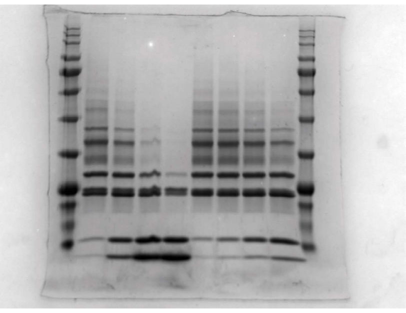

Fig. 2D)

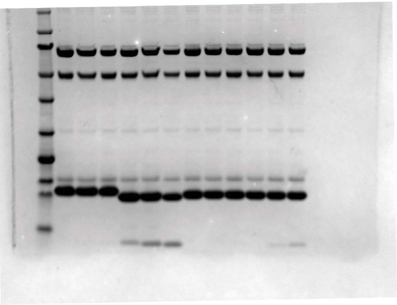

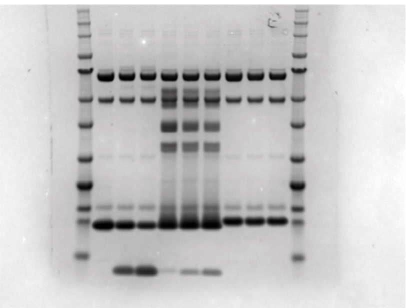

Fig. 2E)

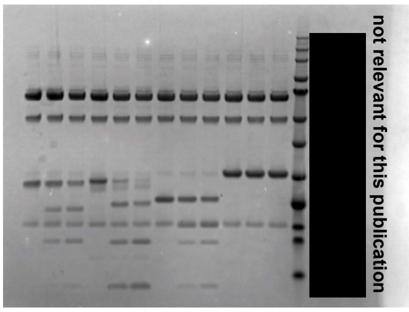

Fig. 2F)

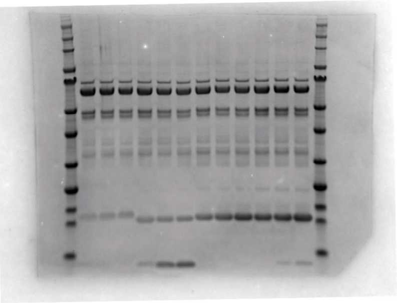

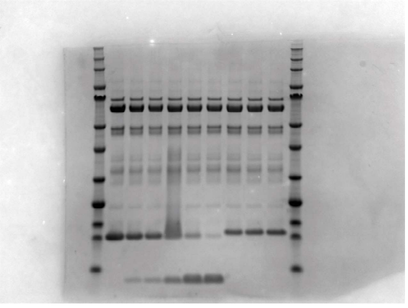

Fig. 2G)

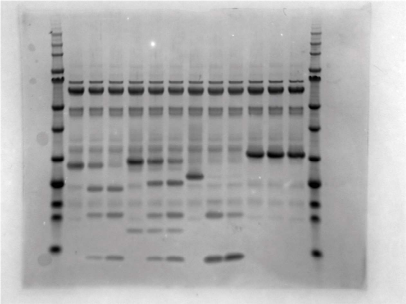

Fig. 2H)

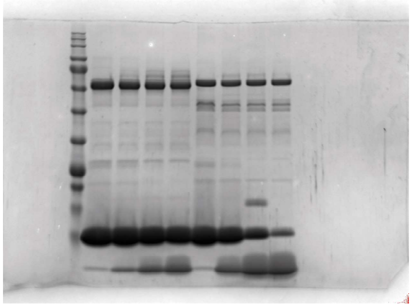

Fig. 3F)

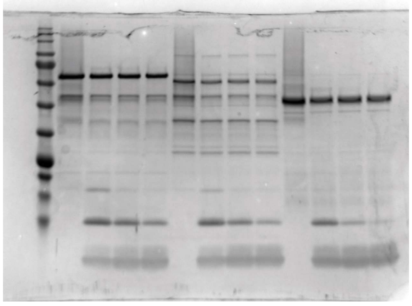

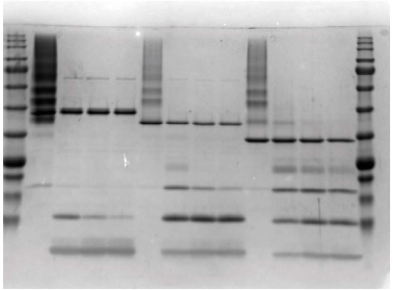

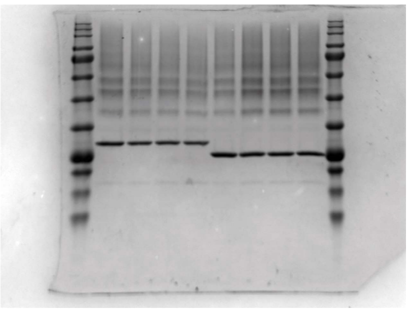

Fig. 4D)

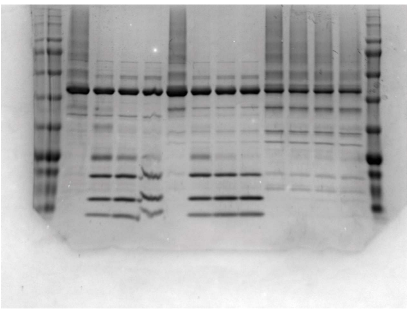

Fig. 4E)

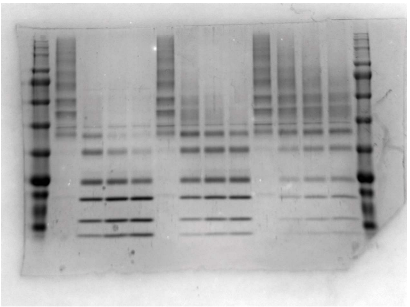

Fig. 4G)

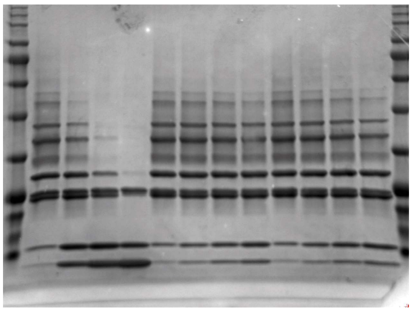

Fig. 5C)

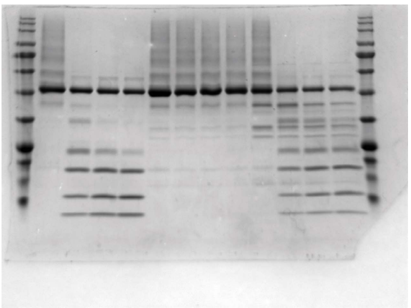

Fig. 5E)

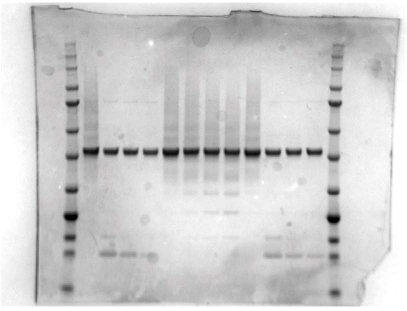

Fig. 5G)

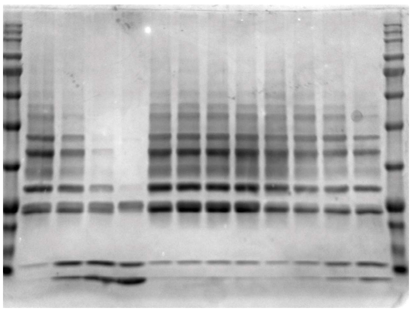

Fig. 5H)

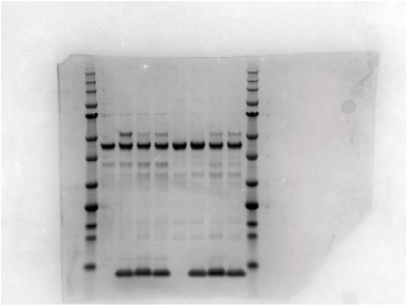

Fig. 6A)

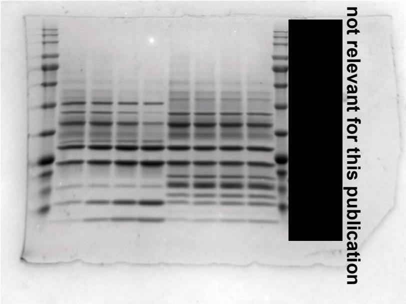

Fig. 6B)

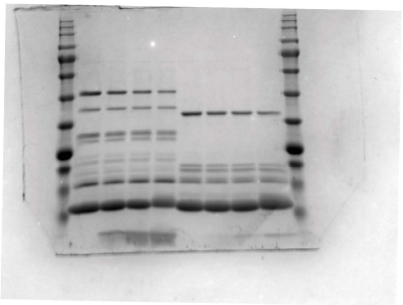

Fig. 6D)

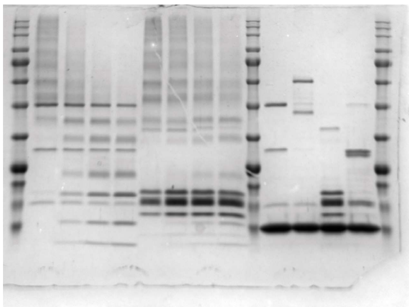

Fig. 6E)

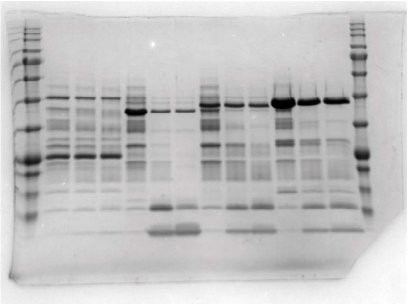

Fig. 6G)

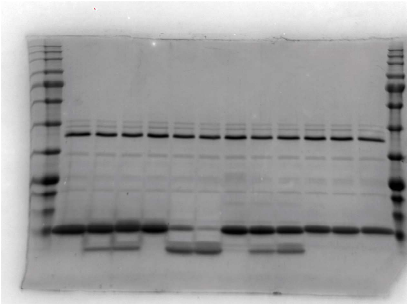

Fig. 7B)

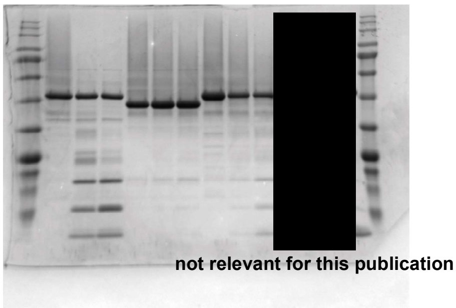

Fig. 7E)

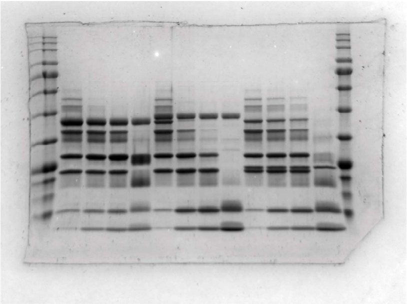

Fig. 7F)

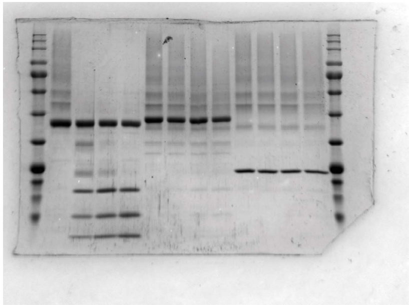

Fig. S1C)

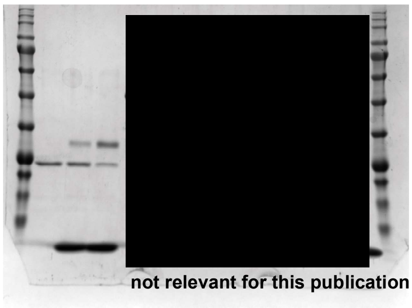

not relevant for this publication

Fig. S2B)

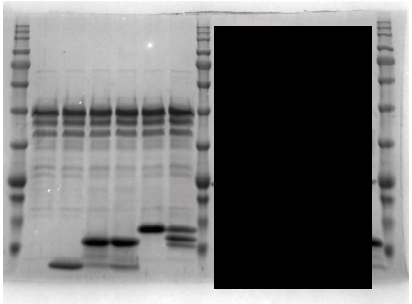

not relevant for this publication

Fig. S2E)

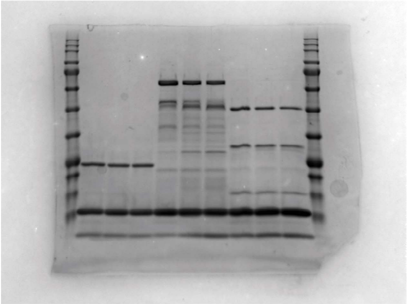

Fig. S2F)

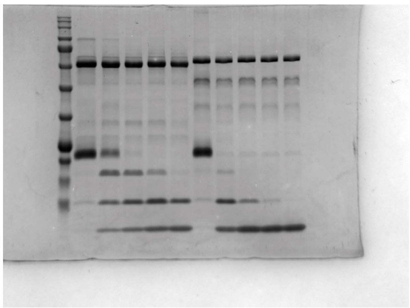

Fig. S2G)

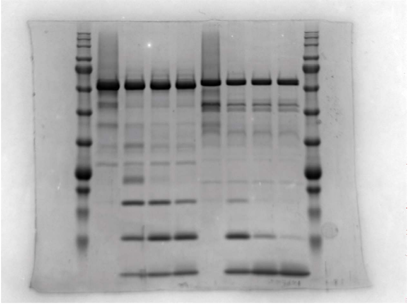

Fig. S2H)

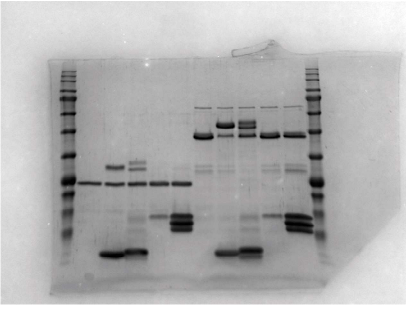

Fig. S2I)

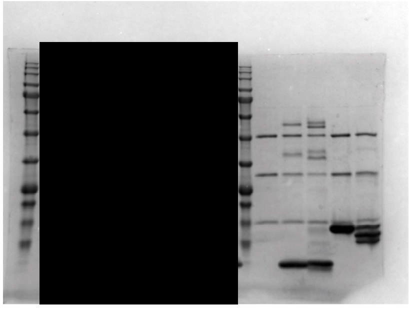

not relevant for this publication

Fig. S3C)

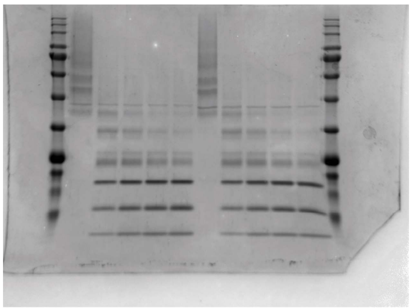

Fig. S3D)

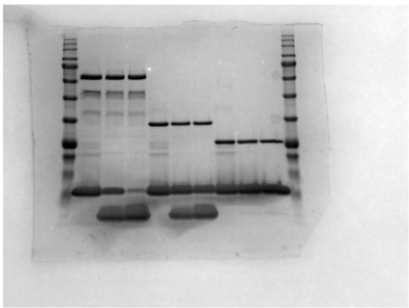

Fig. S3F)

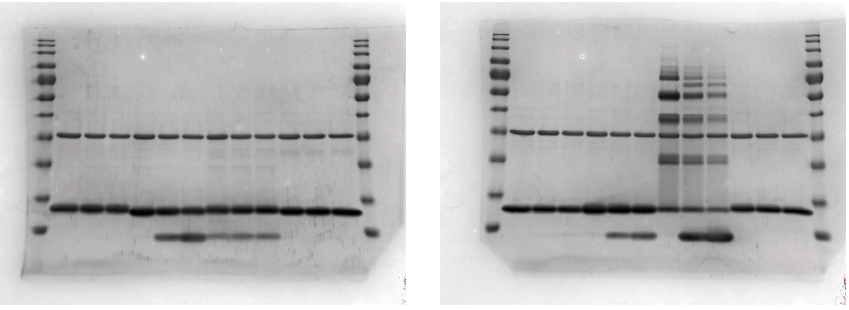

Fig. S3G)

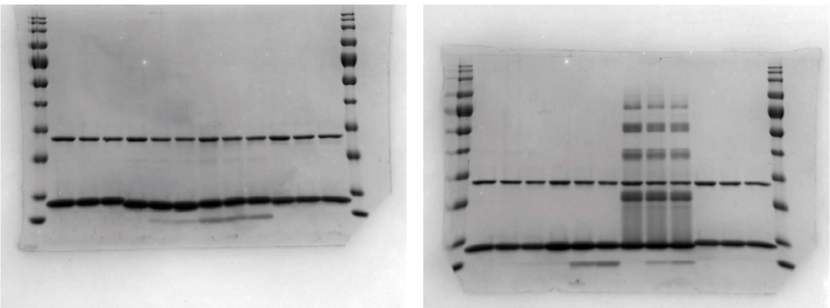

Fig. S4D)

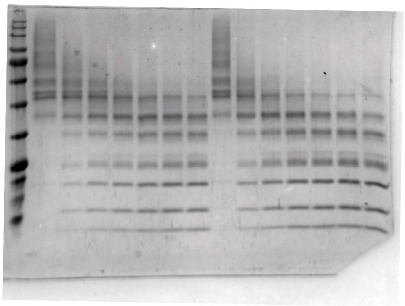

Fig. S5A)

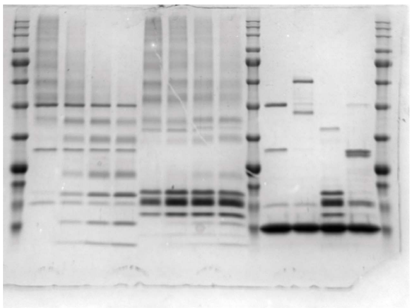

Fig. S5C)

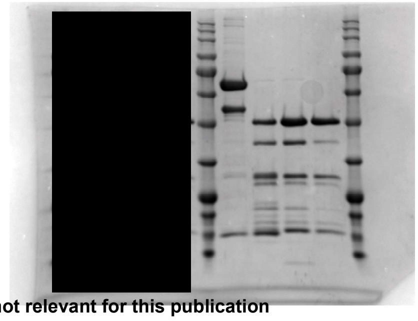

not relevant for this publication

Fig. S56)

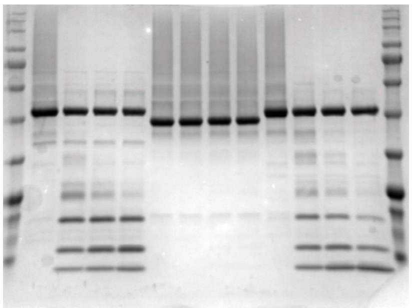

Fig. S6D)

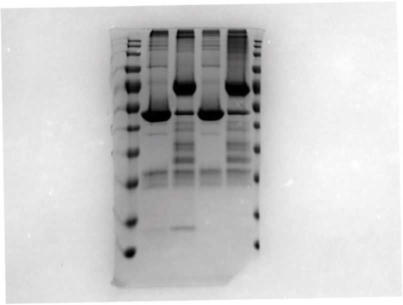

Fig. S6F)

not relevant for this publication

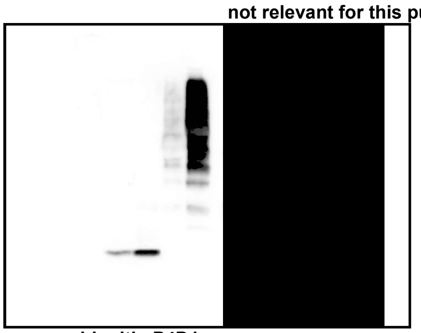

α-ubiquitin P4D1
